# Impact of Treatment Sequencing on Overall Survival in Patients with Transplant-Ineligible Newly Diagnosed Myeloma

**DOI:** 10.1093/oncolo/oyad053

**Published:** 2023-04-01

**Authors:** Rafael Fonseca, Thierry Facon, Mahmoud Hashim, Sandhya Nair, Jianming He, Eric Ammann, Annette Lam, Mark Wildgust, Shaji Kumar

**Affiliations:** Hematology, Mayo Clinic in Arizona, Phoenix, AZ, USA; Hematology, Lille University Hospital, Lille, France; Modeling, Janssen Pharmaceutica NV, Beerse, Antwerp, Belgium; Modeling, Janssen Pharmaceutica NV, Beerse, Antwerp, Belgium; Market Access Analytics, Janssen Pharmaceutica NV, Beerse, Antwerp, Belgium; Market Access Analytics, Janssen Pharmaceutica NV, Beerse, Antwerp, Belgium; Market Access Analytics, Janssen Pharmaceutica NV, Beerse, Antwerp, Belgium; Medical Affairs, Janssen Global Services, Raritan, NJ, USA; Hematology, Mayo Clinic Rochester, Rochester, MN, USA

**Keywords:** antineoplastic agents/therapeutic use, clinical decision-making, multiple myeloma/therapy, outcome assessment, healthcare/methods

## Abstract

**Background:**

Because patients with newly diagnosed multiple myeloma (NDMM) do not always receive any treatment beyond first-line (1L) therapy, it is imperative that patients receive the best treatment in the 1L setting. However, the optimal initial treatment remains to be identified. We performed a clinical simulation to assess potential outcomes with different treatment sequences.

**Patients and Methods:**

We used a partitioned survival model to compare overall survival (OS) with (1) daratumumab, lenalidomide, and dexamethasone (D-Rd) in 1L followed by a pomalidomide- or carfilzomib-based regimen in second line (2L) versus (2) bortezomib, lenalidomide, and dexamethasone (VRd) in 1L followed by a daratumumab-based regimen in 2L versus (3) lenalidomide and dexamethasone (Rd) in 1L followed by a daratumumab-based regimen in 2L. Probabilities of transition between health states (1L, 2L+, and death) were based on published clinical data and real-world data from the Flatiron Health database. The proportion of patients discontinuing treatment after 1L (attrition rates) in the base case was estimated with a binomial logistic model using data from the MAIA trial.

**Results:**

Using D-Rd in 1L conferred a longer median OS compared with delaying daratumumab-based regimens until 2L after VRd or Rd, respectively (8.9 [95% CrI 7.58-10.42] vs. 6.92 [5.92-8.33] or 5.75 [4.50-7.25] years). Results of scenario analyses were consistent with the base case.

**Conclusion:**

Our simulation, which incorporates clinically representative treatments and attrition rates, supports the use of D-Rd as initial therapy, rather than delaying the use of daratumumab until later lines of therapy, in patients with transplant-ineligible NDMM.

## Teaser

Optimal initial treatment for newly diagnosed multiple myeloma in patients ineligible for autologous stem cell transplant remains to be identified. This article assesses potential outcomes with different treatment sequences.

**Implications for Practice**

The optimal treatment sequence for patients with multiple myeloma has not yet been identified. In the absence of randomized clinical trials comparing treatment sequences, simulations can be used to explore the clinical value of different scenarios. Our analysis suggests using daratumumab, lenalidomide, and dexamethasone first, followed by a carfilzomib- or pomalidomide-containing regimen, which confers over 2 years of additional OS gain compared with using bortezomib, lenalidomide, and dexamethasone first (or lenalidomide and dexamethasone first) followed by a daratumumab-containing regimen. This OS gain is especially valuable in transplant-ineligible patients who may not receive treatment beyond initial therapy.

## Introduction

Recent treatment advances have improved overall survival (OS) in patients with multiple myeloma (MM); however, MM remains a mostly incurable malignancy^1-3^ for which treatments generally become less effective with each successive line of therapy (LOT).^4-6^ The rapidly evolving treatment landscape has raised questions about the optimal treatment sequence in patients with newly diagnosed multiple myeloma (NDMM), especially those who are transplant ineligible (TIE).

Studies have shown that while most patients with NDMM receive first-line (1L) treatment, 20.5%-52.9% of patients may not receive any treatment beyond 1L.^5-9^ Reasons for this may include patient characteristics (eg, age, performance status, and comorbidities)^4,7,9^ and limited access to certain treatment options owing to country-specific reimbursement policies.^9^ The proportion of patients who do not move on to subsequent LOT is typically referred to as the “attrition rate.” Importantly, attrition rates generally increase with each successive LOT^4-6,9^ and are higher among TIE patients.^7^ As many patients will not have an opportunity to receive subsequent therapy with later LOT, achieving the longest possible ­progression-free survival (PFS) upfront is critical, as this likely extends OS. Importantly, this prolonged survival provides additional time to potentially take advantage of new therapies currently in development (eg, chimeric antigen receptor T-cell therapy or bispecific antibodies).

Commonly used 1L treatment regimens in these patients include the combination of lenalidomide and dexamethasone (Rd); bortezomib, lenalidomide, and dexamethasone (VRd); and daratumumab, lenalidomide, and dexamethasone (D-Rd), all of which are recommended as primary therapy for non-transplant candidates by the National Comprehensive Cancer Network (NCCN) with Category 1 evidence.^10^ The SWOG S0777 trial compared VRd and Rd in patients with NDMM for whom immediate autologous stem cell transplant (ASCT) was not intended and demonstrated a significant OS benefit with VRd versus Rd (HR 0.709; 95% CI, 0.543-0.926; *P* = .0114).^11^ Many of the patients included in SWOG S0777 were transplant eligible but chose to defer transplant until later. Within the subset of patients ≥65 years old (ie, those who can be considered a proxy for TIE patients), significant OS benefit was not yet demonstrated (HR 0.769; 95% CI, 0.520-1.138; *P* = .168).^11^ In the MAIA trial, which compared D-Rd versus Rd, all patients were TIE, and D-Rd demonstrated a significant PFS and OS benefit versus Rd (PFS HR 0.53; 95% CI, 0.43-0.66; *P* < .0001 and OS HR 0.68; 95% CI, 0.53-0.86; *P* = .0013).^12^

In the absence of long-term head-to-head clinical trials comparing different treatment sequences (some trials are now ongoing), post hoc clinical trials and real-world data can be used to develop clinical simulations to explore different scenarios. Two previously published simulations^13,14^ suggested that delaying daratumumab until later LOT is a viable treatment strategy; however, these models have important limitations that need to be considered, including the use of treatment pathways that do not reflect current clinical practice or clinical guidelines. These simulations also rely on clinical inputs for second-line (2L) treatment and beyond that are not representative of 1L TIE patients at relapse who need 2L treatment. Moreover, these simulations do not take into account attrition rates between LOT. To understand the likely outcomes of different treatment approaches typically used in real-world clinical practice, we developed a clinical simulation including attrition rates and contemporaneous data on outcomes with different therapies by LOT to explore outcomes in patients with TIE NDMM.

## Methods

### Analysis Framework and Model Structure

We used a clinical simulation to simulate OS with different treatment sequences in the treatment of TIE NDMM. The clinical simulation was based on a partitioned survival model that estimates the proportion of a cohort in each health state using parametric survival equations.^15^

OS was assessed over a 15-year time horizon following the initiation of 1L therapy. Median OS and 5-, 10-, and 15-year survival rates were estimated. Survival was adjusted for general population mortality using the US Life Table from the National Vital Statistics Report.^16^

**Figure 1.**
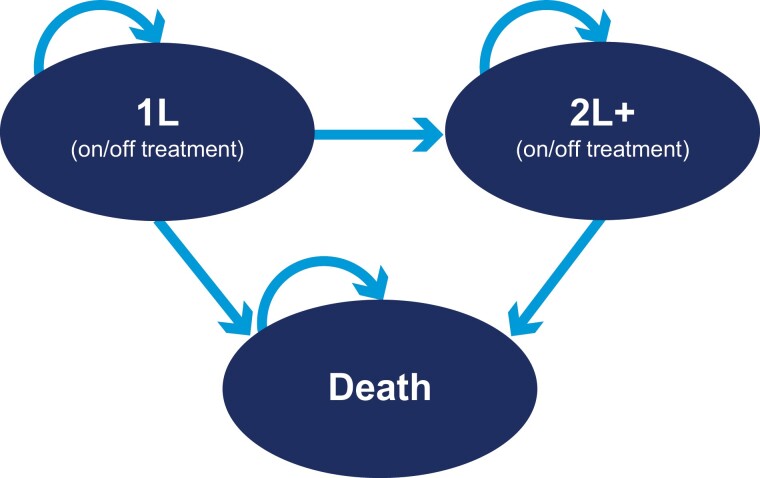
Design of the health state transition model. Abbreviations: 1L, first line; 2L, second line.

Three health states were included (Fig. 1). Patients in the 1L (on/off treatment) health state were on 1L treatment or may have stopped without initiating a 2L treatment. Patients could transition to 2L+ (on/off treatment) or death or remain in this state. Patients in the 2L+ (on/off treatment) health state were on 2L+ treatment or may have stopped without initiating a subsequent LOT. Patients could remain in this state or their condition could worsen resulting in death. The third health state was death, which is an absorbing state. The model cycle was 1 month.

Treatment sequences for third-line and beyond were not explicitly considered because (1) the objective aim of the analysis was to compare sequences in the first-2 lines, (2) including more LOT requires the use of a more sophisticated and data-intensive model, and (3) efficacy of these treatments is incorporated indirectly by including OS on 2L.

### Treatment Regimens

**Figure 2.**
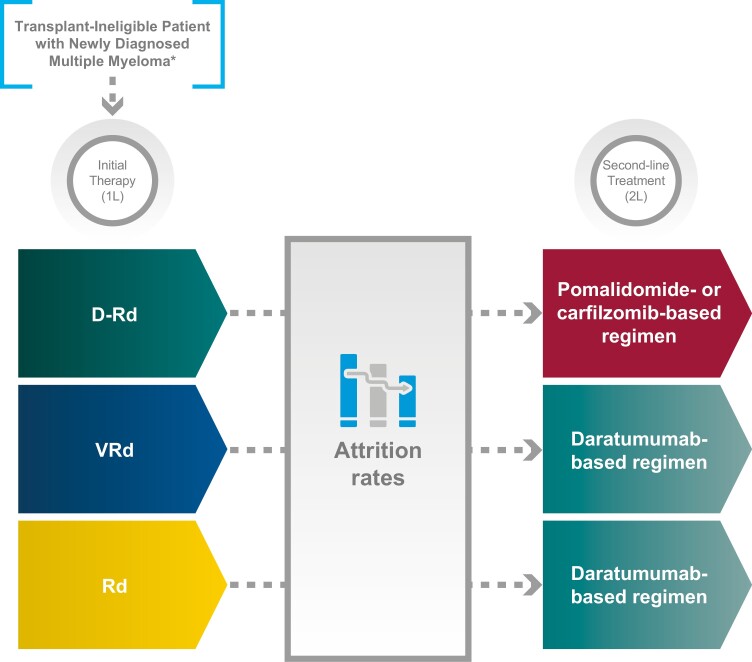
Treatment sequences included in the clinical simulation. Abbreviations: D-Rd, daratumumab, lenalidomide, and dexamethasone; Rd, lenalidomide and dexamethasone; VRd, bortezomib, lenalidomide, and dexamethasone.

Three treatment sequences representative of current practice and based on NCCN^10^ and European Society of Medical Oncology treatment guidelines^2^ were included in the analysis (Fig. 2): D-Rd in 1L followed by pomalidomide- or ­carfilzomib-based regimens in 2L, VRd in 1L followed by daratumumab-based regimens in 2L, and Rd in 1L followed by daratumumab-based regimens in 2L.

### Patient Population

The clinical simulation relied on published clinical trial data and data from the Flatiron Health electronic health record-derived de-identified database. Patients entering the clinical simulation were assumed to match the MAIA trial patient population, with a diagnosis of TIE NDMM and an average age of 74.1 years. Given this, a 15-year horizon represents a reasonable projection of patients’ lifetime. The Flatiron Health database includes longitudinal real-world data from over 280 primarily community-based oncology practices across the US. Patients from the Flatiron database initiating 2L were included if they had never received ASCT and were >65 years old at the start of 1L treatment. Patients initiating a daratumumab-based treatment regimen as 2L therapy were used to characterize outcomes associated with 2L daratumumab-based therapy. Patients initiating a ­carfilzomib- or pomalidomide-based regimen (except in combination with daratumumab) were used to characterize outcomes associated with 2L carfilzomib- or pomalidomide-based therapy. Details regarding the specific regimens received in 2L are shown in Supplementary Table S1. These criteria were applied irrespective of 1L treatment. Having the specific pomalidomide/carfilzomib curves after 1L daratumumab was not possible because limited real-world follow-up data after frontline daratumumab were available. Having the specific daratumumab curves after 1L VRd was possible but had no impact on the estimated OS.

### Transition Probabilities

To inform 1L, 1-month transition probabilities were derived from time to next treatment (TTNT; defined as time to next treatment line or death), PFS, and OS data from the MAIA trial^12,17^ and SWOG S0777 (based on a published network meta-analysis^18^). Real-world data from the Flatiron Health database were used to inform 2L.

Typically, PFS is used to model the transition from 1L (on/off treatment) to 2L+ (on/off treatment). However, for D-Rd and Rd, time spent in the 1L on/off treatment health state was based on TTNT curves derived from the MAIA trial using standard extrapolation techniques. By using TTNT, we were able to explicitly incorporate the proportion of patients who would die before receiving 2L treatment into the model (see information on attrition rates below). TTNT curves for VRd were derived by applying PFS hazard ratios from a published network meta-analysis.^18^ A TTNT HR for VRd was not available; however, in similar studies, the HRs for TTNT and PFS are usually very similar (eg, in MAIA, PFS HR was 0.53 [95% CI, 0.43-0.66] and TTNT HR was 0.47 [95% CI, 0.37-0.59]^12^). Therefore, PFS HR was used as a proxy in the current model.

Time spent in the 2L on/off treatment health state was based on real-world OS curves derived from the Flatiron Health database using standard extrapolation techniques. To more accurately reflect the MAIA patient population, patients were included if they had never received ASCT, were >65 years old at the start of 1L treatment, and had initiated a pomalidomide-, carfilzomib-, or daratumumab-based regimen in 2L. OS associated with pomalidomide- or carfilzomib-based regimens in 2L was estimated separately from OS associated with daratumumab-based regimens in 2L. Real-world data were used instead of clinical trial data because randomized controlled trials (RCTs) in relapsed/refractory MM (RRMM) include patients who are not reflective of those who would progress after 1L treatment in MAIA. Specific differences in baseline characteristics for patients in MAIA versus those in RCTs in RRMM are summarized in Supplementary Table S2.

In our clinical simulation, a fixed proportion of patients leaving the 1L (on/off treatment) health state would transition to the death health state without receiving 2L treatment. This proportion (the attrition rate) was based on the MAIA trial. Several methods to estimate attrition rates were explored. These are described in the Supplementary Material, and the final model used to estimate attrition rates is shown in Supplementary Table S3. In the base case, attrition rates were calculated as the predicted number of death events divided by the total number of patients (D‑Rd 29.9% [110/368]; Rd 24.4% [90/369]). Given the absence of data for VRd, we used the pooled overall estimate from MAIA ([200/737] 27.1%) in the VRd arm.

### Scenario Analyses

In a sensitivity analysis used to confirm the base case, attrition rates were assumed to be the same for all treatments and were calculated using 2 different and simple approaches, both based on the MAIA trial (although they could be different in everyday practice). The first approach resulted in an attrition rate of 58.8%, which was obtained by calculating the proportion of the 729 patients who received ≥1 dose of study treatment who did (*n* = 300, 41.2%) and did not (*n* = 429, 58.8%) receive subsequent therapy. Because this rate included censored patients in the denominator, it is possible that this is an overestimation, although it is similar to other published reports.^7,8^ The second approach excluded censored patients (*n* = 325), resulting in a more conservative attrition rate of 27.2%. It is also important to note that, at a median follow-up of 56.2 months, 222 patients remained on study treatment (155 on D-Rd and 67 on Rd) in MAIA.^12^

We also performed a scenario analysis using the PFS HR of VRd versus Rd based on the PEGASUS study,^19^ an indirect treatment comparison of D-Rd versus VRd and Rd that used data from the MAIA trial and real-world data from the Flatiron Health database. Additionally, several scenario analyses were conducted using different parametric distributions representing best (highest survival benefits) and worst (lowest survival benefits) options.

A probabilistic sensitivity analysis (PSA) was conducted in which each model input parameter was represented by a standard probability distribution. Within the PSA, values were randomly drawn from the distribution for each parameter to obtain estimates of model outcomes for each treatment sequence. This procedure was repeated 1000 times. The results of the PSA were used to estimate uncertainty around point estimates.

## Results

**Figure 3.**
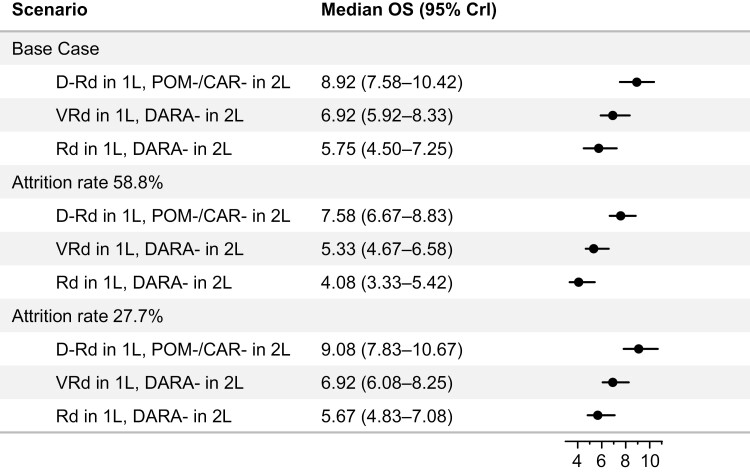
Median OS (95% CrI) for base case and main sensitivity analyses. Abbreviations: CAR, carfilzomib; CrI, credible interval; DARA, daratumumab; D-Rd, daratumumab, lenalidomide, and dexamethasone; OS, overall survival; POM, pomalidomide; Rd, lenalidomide and dexamethasone; VRd, bortezomib, lenalidomide, and dexamethasone.

**Figure 4.**
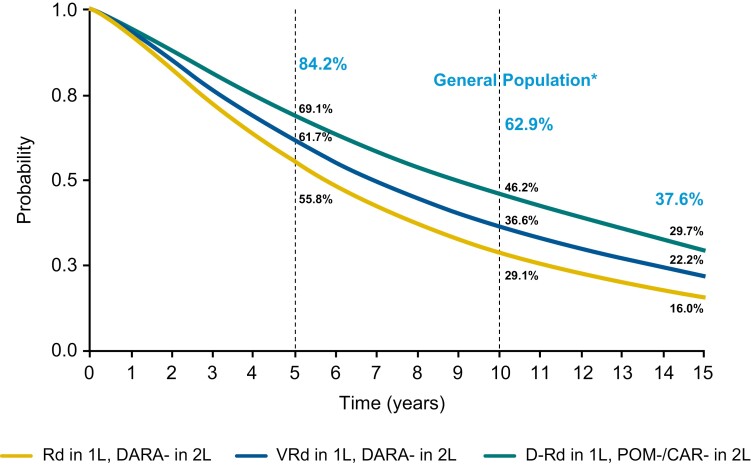
5-, 10-, and 15-year OS rates (base case). ^*^ Median simulated age 86.7 years (US Life Table, National Vital Statistics Reports, vol. 68, No. 7, June 24, 2019). Abbreviations: CAR, carfilzomib; DARA, daratumumab; D-Rd, daratumumab, lenalidomide, and dexamethasone; OS, overall survival; POM, pomalidomide; Rd, lenalidomide and dexamethasone; VRd, bortezomib, lenalidomide, and dexamethasone.

In the base case, the median OS (95% CrI) for D-Rd in 1L followed by a pomalidomide- or carfilzomib-based regimen in 2L was 8.9 (7.58-10.42) years, compared with 6.92 (5.92-8.33) and 5.75 (4.50-7.25) years with VRd and Rd, respectively, in 1L followed by a daratumumab-based regimen in 2L (Fig. 3). This difference translated to an additional 2.0 (95% CrI 0-3.83) and 3.17 (1.0-5.08) years of life compared with VRd or Rd, respectively, in 1L followed by a daratumumab-based regimen in 2L. Additionally, survival rates were highest with the sequence of D-Rd in 1L followed by a pomalidomide- or carfilzomib-based regimen in 2L at 5, 10, and 15 years (Fig. 4).

When an alternate attrition rate of 58.8% was applied in a sensitivity analysis, the median (95% CrI) OS decreased slightly, but somewhat proportionally, compared with the base case for all 3 treatment sequences (7.58 [6.67-8.83], 5.33 [4.67-6.58], and 4.08 [3.33-5.42] years for sequences using D-Rd, VRd, and Rd in the first line, respectively; Fig. 3); however, the relative differences between the sequences were consistent with the base case (2.25 [0.83-3.67] and 3.5 [2.08-4.92] years).

In another sensitivity analysis, the median OS was similar to the base case analysis when an attrition rate of 27.2% was applied (9.08 [7.83-10.67], 6.92 [6.08-8.25], and 5.67 [4.83-7.08] years for sequences using D-Rd, VRd, and Rd in the first line, respectively; Fig. 3). Relative differences between the treatment sequences were also similar (2.17 [0.5-3.75] and 3.42 [1.65-5.00] years).

Results were consistent across the remaining scenario ­analyses. Median (95% CrI) OS ranged from 8.0 (5.67-11.83) to 9.50 (8.42-10.75) years for D-Rd in 1L followed by pomalidomide- or carfilzomib-based regimens in 2L, 6.25 (2.58-4.92) to 7.92 (6.58-9.58) years for VRd in 1L followed by a daratumumab-based regimen in 2L, and 5.42 (1.67-4.00) to 6.50 (4.92-8.58) years for Rd in 1L followed by ­daratumumab-based regimens in 2L (Supplementary Fig. S1). Across all scenario analyses, D-Rd in 1L followed by pomalidomide- or carfilzomib-based regimens in 2L conferred between 1.0 (–1.17 to 3.0) and 2.67 (2.66-7.92) years of life versus VRd in 1L followed by a daratumumab-based regimen in 2L, and between 2.33 (0.67-4.42) and 3.75 (1.92-5.42) years of life versus Rd in 1L followed by a daratumumab-based regimen in 2L. In all scenario analyses, survival rates at 5, 10, and 15 years were highest with D-Rd in 1L followed by pomalidomide- or carfilzomib-based regimens in 2L (Supplementary Table S4).

## Discussion

In patients with NDMM, especially those who are the most vulnerable (those who are TIE), using an optimized clinical treatment approach will likely increase OS, while also affording the best chance to access novel therapies still in development. With the rapid clinical development and approval of new therapies for patients with myeloma, randomized head-to-head clinical studies are not available for every potential clinical approach. Hence, we propose this simulation to explore optimal treatment sequences that will confer a deep and durable response, ultimately prolonging long-term survival. Using a clinical simulation, we showed that using D-Rd in 1L followed by pomalidomide- or carfilzomib-based therapies in 2L may provide 2.0 additional years of life compared with using VRd in 1L followed by daratumumab-based treatment in 2L. Similarly, using D-Rd in 1L may provide 3.2 additional years of life compared with using Rd in 1L followed by daratumumab-based treatment in 2L. This increase in OS was consistent and independent of varying attrition rates between 1L and 2L. While we understand that the only definitive answer will come from a randomized phase III trial, the answer is still many years away. Furthermore, we believe the simulation is instructive given that these regimens are supported by NCCN^10^ (Grade 1 evidence), mSMART,^20^ and ESMO guidelines.^2^

Two previously published models explored which first-line treatment might provide the best long-term outcomes for patients, suggesting that saving daratumumab until later lines of therapy is a viable treatment strategy. Patel et al^13^ used a Markov model to estimate the cost and utility of different treatment strategies over a lifetime horizon in patients with TIE NDMM. They concluded that using daratumumab in 1L was associated with an improvement of 0.52 quality-adjusted life-years and 0.66 discounted life-years versus using daratumumab in 2L. The median OS observed with their model was 7.50 years for 1L use of daratumumab (similar to the 7.6 years observed in our study) versus 6.75 years when daratumumab was used in later lines. Although Patel et al found better clinical outcomes with early versus later use of daratumumab, they proposed that delaying daratumumab until subsequent LOT might be a reasonable treatment strategy. The life-years associated with saving daratumumab for later use may be overestimated, as many patients may not be treated with subsequent daratumumab due to attrition. Similarly, Blommestein et al^14^ used a discrete event simulation based on data from the Dutch PHAROS registry to evaluate 30 different treatment sequences in patients with NDMM. They suggested that sequences with D-VMP in 1L had an expected OS of 7.5 years. The models used by Patel et al and Blommestein et al have notable limitations that impact their conclusions, including basing conclusions on treatment sequences that are neither commonly used in practice today nor recommended in clinical guidelines and not accounting for attrition rates. Furthermore, their models relied on RCTs in RRMM to inform 2L treatment and beyond, although patients in those studies are not representative of 1L TIE patients at relapse in need of 2L treatment—these patients tend to be younger and may have had a prior ASCT. Finally, neither model adjusted for general population mortality.

Although the Patel et al and Blommestein et al studies were cost-effectiveness studies, our clinical simulation was developed to answer the same clinical question, while addressing the limitations of their respective models. Our simulation was based on a partitioned survival model, which is a commonly used approach in the assessment of long-term outcomes of oncology therapies. The treatment regimens included in our clinical simulation are recommended by treatment guidelines^2^ and are commonly used in clinical practice today. Attrition rates were incorporated into our approach to account for the substantial number of patients who may not receive treatment beyond 1L. In our base case, different attrition rates ranging from 24.4% to 29.9% were applied to each of the 3 treatment sequences. Sensitivity analyses explored additional attrition rates from the MAIA trial that included and excluded censored patients (58.8% and 27.2%, respectively), applied to all 3 treatment sequences. The consistent results achieved with these rates highlight the robustness of our clinical simulation. Finally, inputs for 2L and beyond came from real-world data rather than published trials in RRMM to ensure that patient baseline characteristics such as age and prior ASCT were representative of 1L TIE patients at relapse and in need of 2L treatment. Patients enrolled in RCTs of RRMM are significantly younger than patients in the MAIA trial (median age 65-67 vs. 73 years old), and >56% had prior ASCT. The real-world 2L patient outcomes used as inputs for the model were broadly consistent with other real-world data reported in the literature.^21,22^

Although it was developed to address the limitations observed in the models of Patel et al and Blommestein et al, our simulation does have limitations, and our assumptions require further validation. First, the use of real-world survival data in the 2L setting may result in an underestimation of OS compared with the trial setting. However, being conservative in this manner does not impact the overall conclusion regarding the preferred treatment sequence. Second, in the absence of a long-term follow-up RCT in MM to compare treatment options or predict clinical outcomes, we leveraged heterogeneous data sources to simulate patient treatment trajectory. Because we did not have patient-level data for the SWOG S0777 trial, we estimated the TTNT HR for VRd versus Rd based on the published PFS HR. Although, in general, the point estimators between PFS and TTNT are consistent, they are seldom identical. Third, our simulation does not account for emerging new treatments in MM, which may allow some patients with better prognosis to receive more effective downstream treatment. Fourth, we assumed that attrition is independent of follow-up period.

Significant advances have been made in the myeloma setting in the last decade; however, we continue to face challenges in treating patients who are elderly, frail, have comorbidities, and/or are not eligible for transplant. Optimizing their initial treatment will likely provide the best quality and quantity of life. However, with the rapid development of new therapies, head-to-head trials comparing different treatment sequences are not available. Moreover, there is a growing body of evidence demonstrating the substantial proportion of patients who do not receive therapy beyond 1L, suggesting that saving treatments until later LOT is an ineffective strategy that does not optimize long-term outcomes. Both D-Rd and VRd are commonly used treatment regimens in patients with NDMM, but in the absence of comparative trials it may be unclear which therapy to choose. Given the PFS and OS benefits observed with D-Rd in 2L in the POLLUX trial, using VRd as initial therapy may seem like a rational approach. However, it is important to bear in mind that the longest PFS and OS data seen to date in patients with TIE NDMM come from the MAIA trial,^12^ and only daratumumab-containing regimens (D-VMP in ALCYONE^23^ and D-Rd in MAIA^12^) have been shown to have significant OS benefit in patients with TIE NDMM. A previous clinical sequencing simulation study demonstrated good OS in TIE patients treated with 1L D-Rd or D-VMP.^24^ Our clinical simulation found that using daratumumab in 1L, rather than delaying until 2L, provided clinically meaningful benefits to patients, including OS rates that begin to approach those of the general population. It also seems implicit and logical that using the regimen with the longest PFS (ie, D-Rd, as observed in MAIA) first would translate into better OS. In addition, prolonged PFS may also increase the probability that more patients will ultimately have the opportunity to benefit from treatments currently still in development. Lastly, although this was not the subject of our study, the absence of enduring toxicities, namely peripheral neuropathy as seen with bortezomib, further favors the use of daratumumab in the frontline setting. Based on the totality of available data and the results of our clinical simulation, it would appear that using D-Rd first is the optimal initial treatment regimen for patients with TIE NDMM.

## Supplementary Material

oyad053_suppl_Supplementary_Material

## Data Availability

The data sharing policy of Janssen Pharmaceutical Companies of Johnson & Johnson is available at https://www.janssen.com/clinicaltrials/transparency. Requests for access to the MAIA study data can be submitted through Yale Open Data Access (YODA) Project site at http://yoda.yale.edu. SWOG S0777 data can be accessed at the National Cancer Institute NCTN/NCORP Archive at https://nctn-data-archive.nci.nih.gov/. The deidentified data originating from Flatiron Health, Inc., may be made available upon request, and are subject to a license agreement with Flatiron Health; interested researchers should contact DataAccess@FH.com to determine licensing terms.
