# Supplementary material for: Impact of Treatment Sequencing on Overall Survival in Patients with Transplant-Ineligible Newly Diagnosed Myeloma
Source: Oncologist. 2023 Apr 1;28(5):e263–9. doi: 10.1093/oncolo/oyad053 (PMC10166176; doi:10.1093/oncolo/oyad053)
Supplement: oyad053_suppl_Supplementary_Material [file oyad053_suppl_supplementary_material.pdf]

Supplementary Material

Attrition Rates

In the base case, a binomial logistic model was used to predict the likelihood of receiving 2L treatment. The model was fitted on patients who had events (either start of 2L or death). As these are post-randomization events, we included the following baseline characteristics in the initial model: assigned treatment (D-Rd vs Rd), sex (male vs female), age (<75 vs ≥75 years), race (white vs others), region (North America vs others), ISS stage (I and II vs III), baseline renal function (creatinine clearance ≤60 vs >60 mL/min), MM type (IgG vs others), cytogenetic profile (high risk and unknown vs standard risk), baseline hepatic function (impaired vs normal), ECOG performance status (0 and 1 vs ≥2), and time since diagnosis in months. Additionally, we considered the observed confirmed best response (≥CR vs others). TTNT was not included as it is not mature for censored patients and, therefore, cannot be used for predictions.

A likelihood ratio test was performed to assess the impact of each baseline characteristic on the model. If the *P*-value for the result was <.05, the variable was considered to contribute significantly to the model. The variables found to be significant contributors to the current model were age, ISS stage, ECOG performance status, and best confirmed response. All other variables were removed from the final model. Using the reduced model (Supplementary Table S2), we predicted the probability of receiving 2L treatment for censored patients overall and per treatment arm. Patients who were younger (<75 years old), fit (ISS stage I and ECOG performance status > 2), and who had not achieved ≥CR were more likely to receive a subsequent LOT.

Supplementary Table S1. Daratumumab-, carfilzomib, and pomalidomide-containing regimens used as 2L therapy in patients from the Flatiron database.

**Regimen, *n* (%)**	**Daratumumab (*N* = 363)**	**Carfilzomib (*N* = 302)**	**Pomalidomide (*N* = 253)**
Abiraterone, bortezomib, daratumumab, and dexamethasone	1 (0.3)	0	0
Abiraterone, bortezomib, daratumumab, dexamethasone, and lenalidomide	1 (0.3)	0	0
Abiraterone, daratumumab, dexamethasone, and lenalidomide	1 (0.3)	0	0
Abiraterone, dexamethasone, ixazomib, and pomalidomide	0	0	1 (0.4)
Abiraterone, dexamethasone, and pomalidomide	0	0	2 (0.8)
Abiraterone and pomalidomide	0	0	1 (0.4)
Bortezomib, carfilzomib, dexamethasone, and lenalidomide	0	3 (1.0)	0
Bortezomib, carfilzomib, dexamethasone, pomalidomide	0	2 (0.7)	2 (0.8)
Bortezomib, cyclophosphamide, daratumumab, and dexamethasone	1 (0.3)	0	0
Bortezomib, cyclophosphamide, daratumumab/hyaluronidase-fihj, and dexamethasone	1 (0.3)	0	0
Bortezomib, cyclophosphamide, dexamethasone, and pomalidomide	0	0	1 (0.4)
Bortezomib and daratumumab	3 (0.8)	0	0
Bortezomib, daratumumab, and dexamethasone	56 (15.4)	0	0
Bortezomib, daratumumab, dexamethasone, and lenalidomide	16 (4.4)	0	0

Bortezomib, daratumumab, dexamethasone, and melphalan	1 (0.3)	0	0
Bortezomib, daratumumab, dexamethasone, and obinutuzumab	1 (0.3)	0	0
Bortezomib, daratumumab, dexamethasone, and osimertinib	1 (0.3)	0	0
Bortezomib, daratumumab, dexamethasone, and palbociclib	1 (0.3)	0	0
Bortezomib, daratumumab, dexamethasone, and pomalidomide	3 (0.8)	0	0
Bortezomib, daratumumab, dexamethasone, and rituximab	1 (0.3)	0	0
Bortezomib, daratumumab, dexamethasone, and thalidomide	1 (0.3)	0	0
Bortezomib, daratumumab, and pomalidomide	1 (0.3)	0	0
Bortezomib, daratumumab, and prednisone	1 (0.3)	0	0
Bortezomib and daratumumab/hyaluronidase-fihj	1 (0.3)	0	0
Bortezomib, daratumumab/hyaluronidase-fihj, and dexamethasone	9 (2.5)	0	0
Bortezomib, daratumumab/hyaluronidase-fihj, dexamethasone, and lenalidomide	5 (1.4)	0	0
Bortezomib, daratumumab/hyaluronidase-fihj, melphalan, and prednisone	1 (0.3)	0	0
Bortezomib, dexamethasone, ixazomib, and pomalidomide	0	0	2 (0.8)
Bortezomib, dexamethasone, lenalidomide, and pomalidomide	0	0	1 (0.4)
Bortezomib, dexamethasone, melphalan, and pomalidomide	0	0	1 (0.4)
Bortezomib, dexamethasone, and pomalidomide	0	0	32 (12.6)
Bortezomib and pomalidomide	0	0	5 (2.0)
Carfilzomib	0	8 (2.6)	0

Carfilzomib and clinical study drug	0	2 (0.7)	0
Carfilzomib, clinical study drug, dexamethasone, and lenalidomide	0	1 (0.3)	0
Carfilzomib and cyclophosphamide	0	2 (0.7)	0
Carfilzomib, cyclophosphamide, daratumumab, and dexamethasone	1 (0.3)	0	0
Carfilzomib, cyclophosphamide, and dexamethasone	0	9 (3.0)	0
Carfilzomib, cyclophosphamide, dexamethasone, and lenalidomide	0	2 (0.7)	0
Carfilzomib, cyclophosphamide, and pomalidomide	0	1 (0.3)	1 (0.4)
Carfilzomib, daratumumab, and dexamethasone	14 (3.9)	0	0
Carfilzomib, daratumumab, dexamethasone, and lenalidomide	1 (0.3)	0	0
Carfilzomib, daratumumab, dexamethasone, and pomalidomide	3 (0.8)	0	0
Carfilzomib and dexamethasone	0	121 (40.1)	0
Carfilzomib, dexamethasone, and doxorubicin pegylated liposomal	0	1 (0.3)	0
Carfilzomib, dexamethasone, and elotuzumab	0	1 (0.3)	0
Carfilzomib, dexamethasone, elotuzumab, and lenalidomide	0	2 (0.7)	0
Carfilzomib, dexamethasone, enzalutamide, and lenalidomide	0	1 (0.3)	0
Carfilzomib, dexamethasone, ixazomib, and lenalidomide	0	2 (0.7)	0
Carfilzomib, dexamethasone, and lenalidomide	0	87 (28.8)	0
Carfilzomib, dexamethasone, lenalidomide, and pomalidomide	0	1 (0.3)	1 (0.4)
Carfilzomib, dexamethasone, melphalan	0	1 (0.3)	0

Carfilzomib, dexamethasone, pomalidomide	0	53 (17.5)	53 (20.9)
Carfilzomib and lenalidomide	0	2 (0.7)	0
Clinical study drug, daratumumab, and dexamethasone	3 (0.8)	0	0
Clinical study drug and pomalidomide	0	0	1 (0.4)
Cyclophosphamide, daratumumab, dexamethasone, and lenalidomide	1 (0.3)	0	0
Cyclophosphamide, dexamethasone, ixazomib, and pomalidomide	0	0	1 (0.4)
Cyclophosphamide, dexamethasone, lenalidomide, and pomalidomide	0	0	1 (0.4)
Cyclophosphamide, dexamethasone, and pomalidomide	0	0	5 (2.0)
Daratumumab	17 (4.7)	0	0
Daratumumab and dexamethasone	57 (15.7)	0	0
Daratumumab, dexamethasone, and elotuzumab	2 (0.6)	0	0
Daratumumab, dexamethasone, and ixazomib	1 (0.3)	0	0
Daratumumab, dexamethasone, ixazomib, and lenalidomide	2 (0.6)	0	0
Daratumumab, dexamethasone, and lenalidomide	70 (19.3)	0	0
Daratumumab, dexamethasone, lenalidomide, and pomalidomide	1 (0.3)	0	0
Daratumumab, dexamethasone, and leuprolide	1 (0.3)	0	0
Daratumumab, dexamethasone, and pomalidomide	42 (11.6)	0	0
Daratumumab, dexamethasone, and thalidomide	1 (0.3)	0	0
Daratumumab and lenalidomide	2 (0.6)	0	0

Daratumumab and pomalidomide	2 (0.6)	0	0
Daratumumab and prednisone	1 (0.3)	0	0
Daratumumab/hyaluronidase-fihj	6 (1.7)	0	0
Daratumumab/hyaluronidase-fihj and dexamethasone	8 (2.2)	0	0
Daratumumab/hyaluronidase-fihj, dexamethasone, and lenalidomide	12 (3.3)	0	0
Daratumumab/hyaluronidase-fihj, dexamethasone, and pomalidomide	8 (2.2)	0	0
Dexamethasone, elotuzumab, and pomalidomide	0	0	12 (4.7)
Dexamethasone, isatuximab-irfc, and pomalidomide	0	0	1 (0.4)
Dexamethasone, ixazomib, and pomalidomide	0	0	7 (2.8)
Dexamethasone, lenalidomide, and pomalidomide	0	0	6 (2.4)
Dexamethasone and pomalidomide	0	0	84 (33.2)
Lenalidomide and pomalidomide	0	0	1 (0.4)
Pomalidomide	0	0	29 (11.5)
Pomalidomide and prednisone	0	0	2 (0.8)

Shaded boxes indicate regimens received by ≥5% of patients.

Supplementary Table S2. Baseline characteristics of patients in MAIA versus those of patients in RCTs of RRMM.

	**MAIA[1] NCT02252172**	**CASTOR[2] NCT02136134**	**POLLUX[3] NCT02076009**	**CANDOR[4] NCT03158688**	**APOLLO[5] NCT03180736**	**ENDEAVOR[6] NCT01568866**	**ASPIRE[7] NCT01080391**
Disease setting	NDMM	RRMM	RRMM	RRMM	RRMM	RRMM	RRMM
Median age, y	73	64	65	64	67	65	64
Median prior LOT, *n*	0	2	1	2	2	2	2
Prior ASCT, %	0	61.2	63.2	57.9	56.3	60.1	56.3

Abbreviations: ASCT, autologous stem cell transplant; LOT, line of therapy; RCT, randomized controlled trial; RRMM, relapsed/refractory multiple myeloma.

Supplementary Table S3. Final model used to estimate attrition rates.

Variable	OR	95% CI	*P*-value
(Intercept)	1.16	0.66-2.04	.599
Age (<75 vs ≥75 y)	2.02	1.28-3.21	.003
ISS stage I vs III	2.74	1.30-5.80	.008
ISS stage II vs III	0.97	0.59-1.60	.917
ECOG performance status 0 vs ≥2	2.92	1.48-5.77	.002
ECOG performance status 1 vs ≥2	1.51	0.87-2.64	.147
≥CR vs other	0.54	0.32-0.90	.019

OR > 1 indicates increased likelihood of receiving a subsequent line of therapy.

Abbreviations: CI, confidence interval; CR, complete response; ECOG, Eastern Cooperative Oncology Group; ISS, International Staging System; OR, odds ratio.

Supplementary Figure S1. Median OS (95% CrI) for additional scenario analyses.

Scenario	Median OS (95% CrI), years
TTNT HR from Durie et al (PEGASUS)	
D-Rd in 1L, POM-/CAR- in 2L	8.92 (7.58–10.42)
VRd in 1L, DARA- in 2L	6.67 (5.75–8.00)
Rd in 1L, DARA- in 2L	5.75 (4.50–7.25)
TTNT extrapolation for both D-Rd and Rd in 1L; log-normal model	
D-Rd in 1L, POM-/CAR- in 2L	9.33 (5.58–12.83)
VRd in 1L, DARA- in 2L	7.00 (4.67–10.67)
Rd in 1L, DARA- in 2L	5.83 (3.92–8.75)
TTNT extrapolation for both D-Rd and Rd in 1L; exponential model	
D-Rd in 1L, POM-/CAR- in 2L	8.00 (5.67–11.83)
VRd in 1L, DARA- in 2L	6.50 (4.67–9.33)
Rd in 1L, DARA- in 2L	5.67 (4.00–8.00)
OS extrapolation for DARA-based regimens in 2L; log-normal model	
D-Rd in 1L, POM-/CAR- in 2L	8.92 (7.58–10.58)
VRd in 1L, DARA- in 2L	7.92 (6.58–9.58)
Rd in 1L, DARA- in 2L	6.50 (4.92–8.58)
OS extrapolation for DARA-based regimens in 2L; gamma model	
D-Rd in 1L, POM-/CAR- in 2L	8.92 (7.58–10.58)
VRd in 1L, DARA- in 2L	6.25 (2.58–4.92)
Rd in 1L, DARA- in 2L	5.42 (1.67–4.00)
OS extrapolation for POM-/CAR-based regimens in 2L; log-logistic model	
D-Rd in 1L, POM-/CAR- in 2L	9.50 (8.42–10.75)
VRd in 1L, DARA- in 2L	6.92 (6.0–8.33)
Rd in 1L, DARA- in 2L	5.75 (4.67–7.25)
OS extrapolation for POM-/CAR-based regimens in 2L; exponential model	
D-Rd in 1L, POM-/CAR- in 2L	8.75 (7.5–10.75)
VRd in 1L, DARA- in 2L	6.92 (5.83–8.33)
Rd in 1L, DARA- in 2L	5.75 (4.58–7.33)
Distribution of POM-/CAR-based regimens in 2L; 100% POM-based after D-Rd	
D-Rd in 1L, POM-/CAR- in 2L	8.92 (7.08–11.50)
VRd in 1L, DARA- in 2L	6.92 (5.92–8.25)
Rd in 1L, DARA- in 2L	5.75 (4.58–7.33)
Distribution of POM-/CAR-based regimens in 2L; 100% CAR-based after D-Rd	
D-Rd in 1L, POM-/CAR- in 2L	8.92 (7.75–10.25)
VRd in 1L, DARA- in 2L	6.92 (5.83–8.25)
Rd in 1L, DARA- in 2L	5.75 (4.50–7.25)

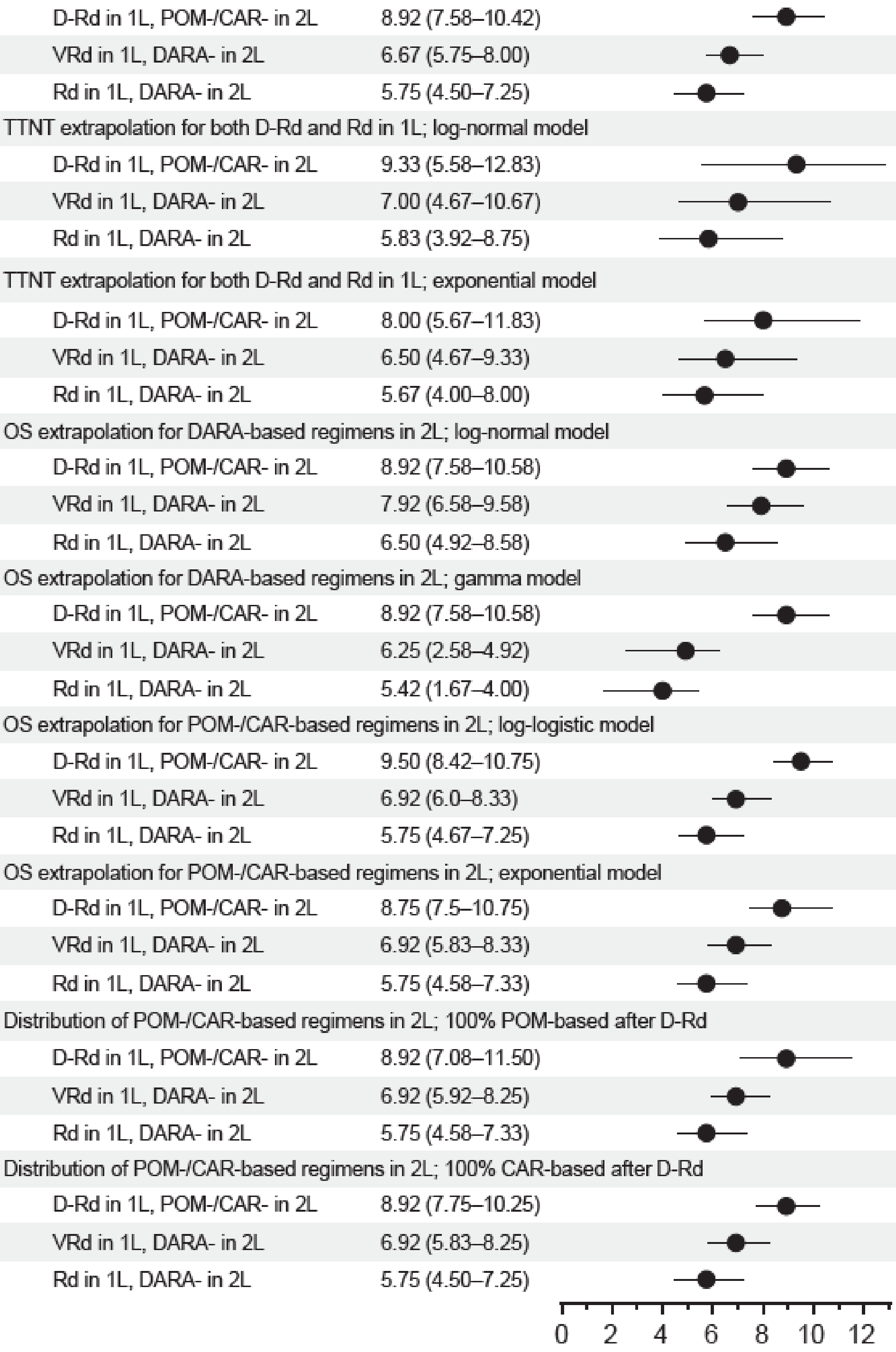

Abbreviations: 1L, first line; 2L, second line; CAR, carfilzomib; CrI, credible interval; DARA, daratumumab; D-Rd, daratumumab, lenalidomide, and dexamethasone; HR, hazard ratio; OS, overall survival; Rd, lenalidomide and dexamethasone; POM, pomalidomide; TTNT, time to next treatment; VRd, bortezomib, lenalidomide, and dexamethasone.

Supplementary Figure S2. Survival rates at (A) 5 and (B) 10 years (scenario analyses).

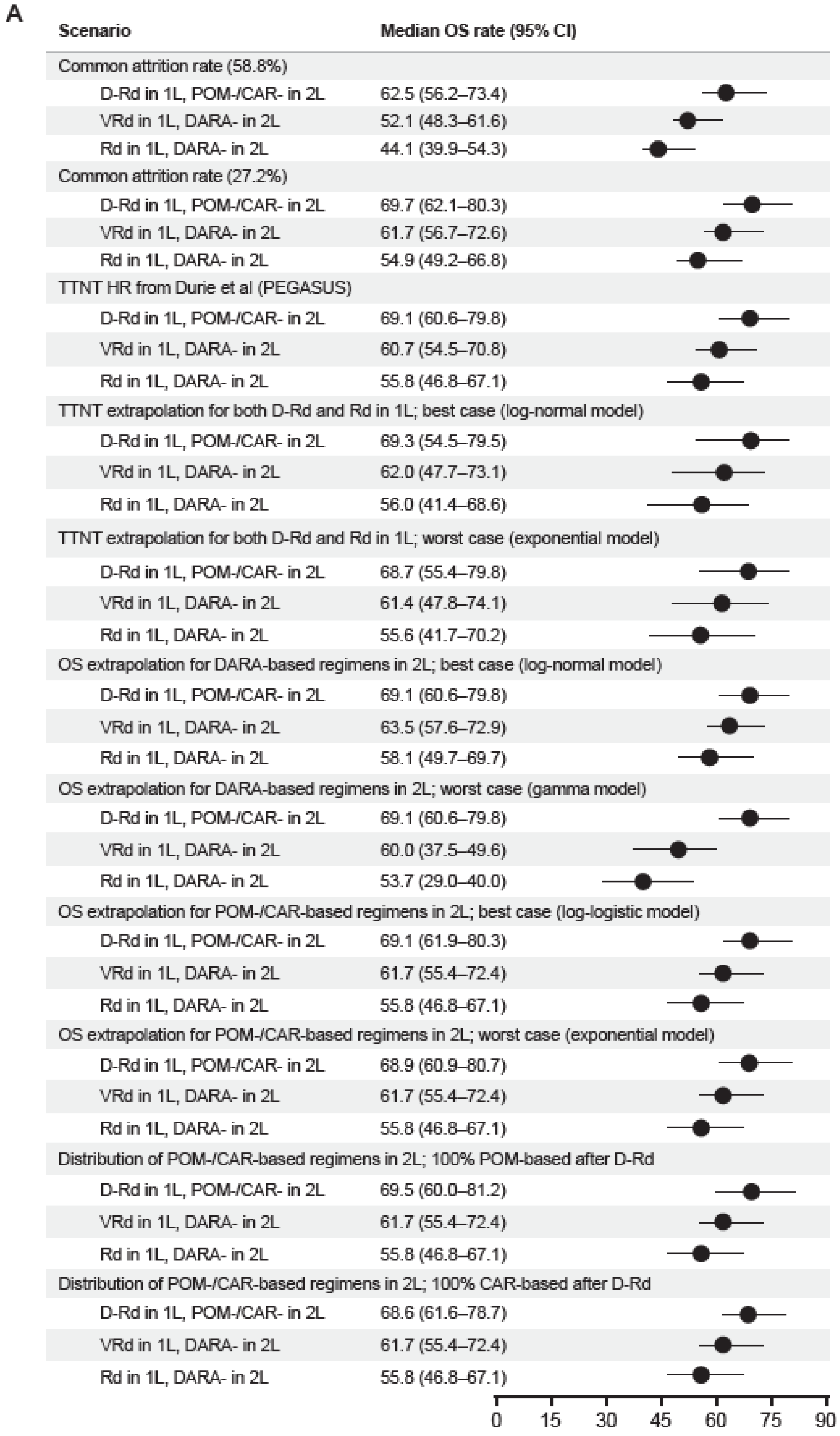

B

Scenario	Median OS rate (95% CI)
Common attrition rate (58.8%)	
D-Rd in 1L, POM-/CAR- in 2L	41.7 (39.6–45.3)
VRd in 1L, DARA- in 2L	30.9 (28.1–34.4)
Rd in 1L, DARA- in 2L	22.8 (20.2–26.2)
Common attrition rate (27.2%)	
D-Rd in 1L, POM-/CAR- in 2L	46.6 (42.9–52.7)
VRd in 1L, DARA- in 2L	36.6 (32.8–41.8)
Rd in 1L, DARA- in 2L	28.6 (24.5–34.0)
TTNT HR from Durie et al (PEGASUS)	
D-Rd in 1L, POM-/CAR- in 2L	46.2 (42.1–51.8)
VRd in 1L, DARA- in 2L	35.4 (31.2–40.8)
Rd in 1L, DARA- in 2L	29.1 (24.2–36.4)
TTNT extrapolation for both D-Rd and Rd in 1L; best case (log-normal model)	
D-Rd in 1L, POM-/CAR- in 2L	47.8 (28.7–60.3)
VRd in 1L, DARA- in 2L	37.3 (20.3–52.3)
Rd in 1L, DARA- in 2L	29.7 (15.7–45.6)
TTNT extrapolation for both D-Rd and Rd in 1L; worst case (exponential model)	
D-Rd in 1L, POM-/CAR- in 2L	40.2 (25.1–57.4)
VRd in 1L, DARA- in 2L	30.7 (17.3–47.1)
Rd in 1L, DARA- in 2L	24.4 (3.4–39.3)
OS extrapolation for DARA-based regimens in 2L; best case (log-normal model)	
D-Rd in 1L, POM-/CAR- in 2L	46.2 (42.1–51.8)
VRd in 1L, DARA- in 2L	43.2 (38.3–48.8)
Rd in 1L, DARA- in 2L	37.0 (30.9–45.2)
OS extrapolation for DARA-based regimens in 2L; worst case (gamma model)	
D-Rd in 1L, POM-/CAR- in 2L	46.2 (42.1–51.8)
VRd in 1L, DARA- in 2L	30.1 (21.6–27.2)
Rd in 1L, DARA- in 2L	21.4 (14.2–18.9)
OS extrapolation for POM-/CAR-based regimens in 2L; best case (log-logistic model)	
D-Rd in 1L, POM-/CAR- in 2L	48.4 (45.1–53.2)
VRd in 1L, DARA- in 2L	36.6 (32.6–42.6)
Rd in 1L, DARA- in 2L	29.1 (24.2–36.4)
OS extrapolation for POM-/CAR-based regimens in 2L; worst case (exponential model)	
D-Rd in 1L, POM-/CAR- in 2L	45.5 (41.4–53.0)
VRd in 1L, DARA- in 2L	36.6 (32.6–42.6)
Rd in 1L, DARA- in 2L	29.1 (24.4–36.4)
Distribution of POM-/CAR-based regimens in 2L; 100% POM-based after D-Rd	
D-Rd in 1L, POM-/CAR- in 2L	46.1 (40.3–55.9)
VRd in 1L, DARA- in 2L	36.6 (32.6–42.6)
Rd in 1L, DARA- in 2L	29.1 (24.2–36.4)
Distribution of POM-/CAR-based regimens in 2L; 100% CAR-based after D-Rd	
D-Rd in 1L, POM-/CAR- in 2L	46.3 (42.7–51.1)
VRd in 1L, DARA- in 2L	36.6 (32.6–42.6)
Rd in 1L, DARA- in 2L	29.1 (24.2–36.4)

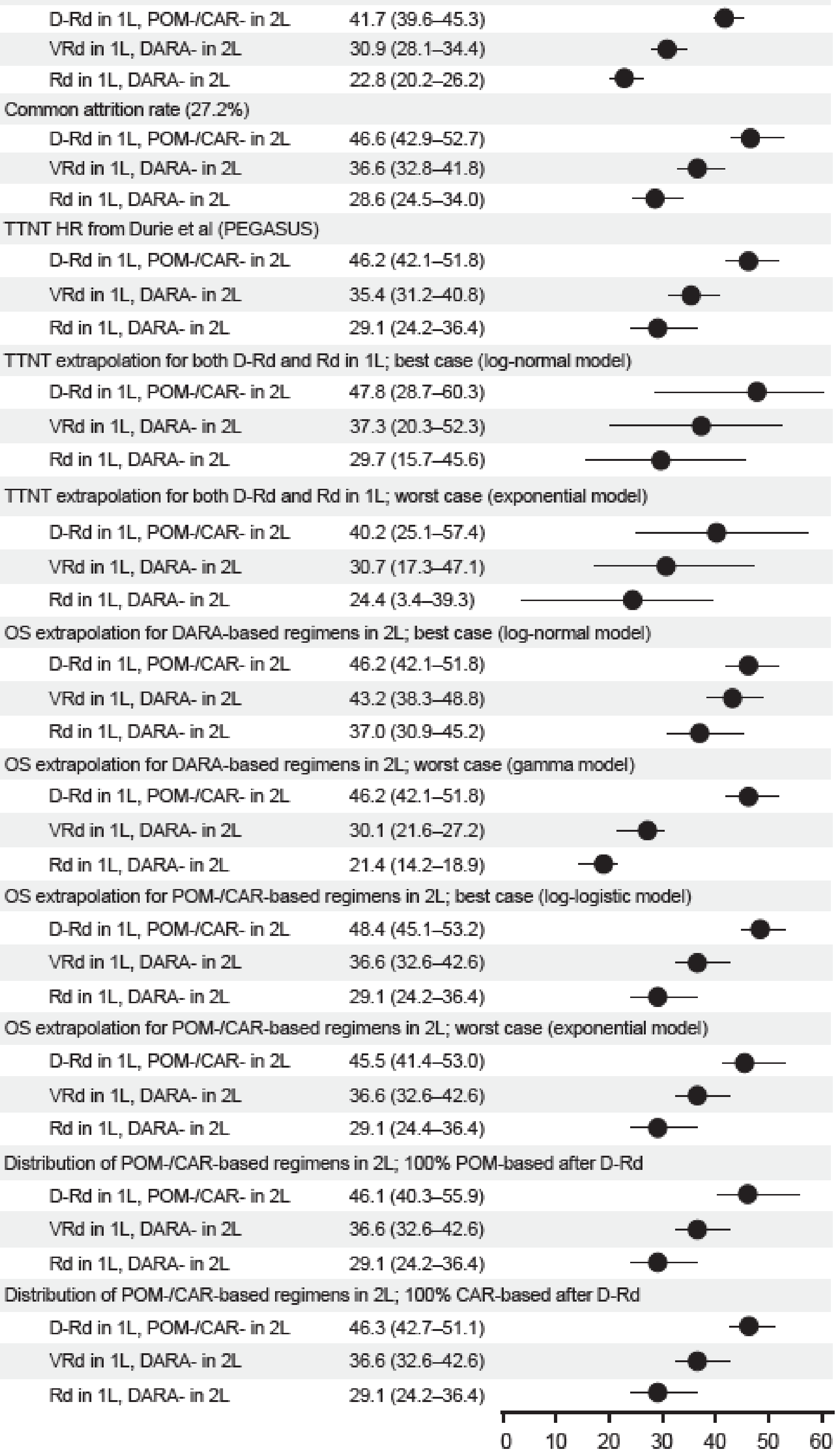

Abbreviations: 1L, first line; 2L, second line; CAR, carfilzomib; CrI, credible interval; DARA, daratumumab; D-Rd, daratumumab, lenalidomide, and dexamethasone; HR, hazard ratio; OS, overall survival; Rd, lenalidomide and dexamethasone; POM, pomalidomide; TTNT, time to next treatment; VRd, bortezomib, lenalidomide, and dexamethasone

References

1 Facon T, Kumar S, Plesner T, et al. Daratumumab plus lenalidomide and dexamethasone for untreated myeloma. N Engl J Med 2019;380:2104-2115.

2 Palumbo A, Chanan-Khan A, Weisel K, et al. Daratumumab, bortezomib, and dexamethasone for multiple myeloma. N Engl J Med 2016;375:754-766.

3 Dimopoulos MA, Oriol A, Nahi H, et al. Daratumumab, lenalidomide, and dexamethasone for multiple myeloma. N Engl J Med 2016;375:1319-1331.

4 Dimopoulos M, Quach H, Mateos MV, et al. Carfilzomib, dexamethasone, and daratumumab versus carfilzomib and dexamethasone for patients with relapsed or refractory multiple myeloma (CANDOR): results from a randomised, multicentre, open-label, phase 3 study. Lancet 2020;396:186-197.

5 Dimopoulos MA, Terpos E, Boccadoro M, et al. Daratumumab plus pomalidomide and dexamethasone versus pomalidomide and dexamethasone alone in previously treated multiple myeloma (APOLLO): an open-label, randomised, phase 3 trial. Lancet Oncol 2021;22:801-812.

6 Dimopoulos MA, Moreau P, Palumbo A, et al. Carfilzomib and dexamethasone versus bortezomib and dexamethasone for patients with relapsed or refractory multiple myeloma (ENDEAVOR): a randomised, phase 3, open-label, multicentre study. Lancet Oncol 2016;17:27-38.

7 Stewart AK, Rajkumar SV, Dimopoulos MA, et al. Carfilzomib, lenalidomide, and dexamethasone for relapsed multiple myeloma. N Engl J Med 2015;372:142-152.
